# Extended‐Spectrum Beta‐Lactamase (ESBL)‐Producing *E. coli* in Livestock and Free‐Roaming Wildlife: A Combined Phenotyping—Whole‐Genome Sequencing One Health Approach

**DOI:** 10.1155/tbed/9907660

**Published:** 2026-08-05

**Authors:** Sheena Conforti, Adamandia Kapopoulou, Claudia Bank, Belinda Köchle, Bahtiyar Yilmaz, Jens Becker

**Affiliations:** ^1^ Eawag, Swiss Federal Institute of Aquatic Science and Technology, Dübendorf 8600, Switzerland; ^2^ Institute of Ecology and Evolution, University of Bern, Bern 3012, Switzerland, unibe.ch; ^3^ Swiss Institute of Bioinformatics, Lausanne 1015, Switzerland, isb-sib.ch; ^4^ Clinic for Ruminants, Vetsuisse-Faculty, University of Bern, Bern 3012, Switzerland, unibe.ch; ^5^ Department of Visceral Surgery and Medicine, Bern University Hospital, University of Bern, Bern 3010, Switzerland, unibe.ch; ^6^ Maurice Müller Laboratories, Department for Biomedical Research, University of Bern, Bern 3008, Switzerland, unibe.ch

**Keywords:** antimicrobials, environment, resistance, selection pressure

## Abstract

Antimicrobial resistance (AMR) in Enterobacterales, particularly extended‐spectrum beta‐lactamase‐producing *Escherichia coli* (ESBL‐Ec), poses a significant public health concern. Widespread antimicrobial use exerts selection pressure, driving the persistence and spread of resistant bacteria in diverse environments. While ESBL‐Ec is well‐documented in clinical and agricultural settings, its presence in animal reservoirs remains poorly understood. This study assessed the prevalence, AMR profiles, and genetic diversity of ESBL‐Ec in Swiss dairy cattle and wildlife. Between 2021 and 2023, 775 samples were analyzed, including rectal swab samples from dairy cows (*n* = 475) and fecal or cloacal swab samples from wildlife (*n* = 300). ESBL‐Ec was detected in 3/475 (0.6%, 95% confidence interval [CI] 0.2%–1.8%) of dairy cattle and 15/300 (5%, 95% CI 3.1%–8.1%) wildlife samples, with higher prevalence in birds (6/99, 6.1%, 95% CI 2.8%–12.6%) than mammals (9/200, 4.5%, 95% CI 2.4%–8.3%). Whole‐genome sequencing (WGS) of 54 ESBL‐Ec isolates identified 28 sequence types (STs) with no overlap between livestock and wildlife, suggesting distinct evolutionary trajectories. Phenotypic testing revealed resistance beyond beta‐lactams, notably against aminoglycosides, tetracyclines, and sulfonamides, whereas all isolates remained susceptible to tigecycline and meropenem. Multidrug resistance (MDR) was prevalent (89%), with bla_TEM-1_ (in dairy cattle), bla_CTX-M55_ and bla_TEM-176_ (in red foxes) widely distributed across Switzerland. Clonal plasmids (IncFIB, IncX1) were detected in both wildlife and dairy cattle, and some animals harbored multiple ESBL‐Ec strains. Despite the low prevalence of plasmids, spatial clustering indicated local persistence in both livestock and wildlife, highlighting potential transmission. The observed presence of clinically relevant ESBL‐Ec in wildlife highlights the need for a One Health approach to tackle the dangers of AMR. Our findings contribute to AMR surveillance by providing baseline data on ESBL‐Ec animal reservoirs and informing the design of strategies to mitigate its environmental spread.

## 1. Introduction

Clinically relevant Enterobacterales pose a significant threat to public health, and their dissemination has garnered considerable attention in recent decades [1, 2]. Of particular concern are isolates carrying extended‐spectrum beta‐lactamase (ESBL) genes, which confer resistance to a wide range of critically important antimicrobial agents, including extended‐spectrum penicillins and third‐ and fourth‐generation cephalosporins. These ESBL‐encoding genes are often located on mobile genetic elements, facilitating their rapid spread across diverse environments, including settings with limited or indirect antimicrobial selection pressures.

Over the past decade, in Europe, ESBL‐producing *Escherichia coli* (ESBL‐Ec) has been detected beyond healthcare settings, notably in livestock, wastewater, and wildlife, underscoring the necessity of a One Health approach to mitigate this public health challenge [3–6]. However, the transmission dynamics of ESBL‐Ec across nonclinical reservoirs and between environmental, animal, and human compartments remain poorly understood, limiting our ability to predict and thus prevent their spread and persistence. Consequently, regular surveillance, including prevalence assessments and phenotypic and genotypic characterizations of ESBL‐Ec, is important to identify sources of dissemination, track emerging resistance patterns, and inform targeted interventions to mitigate their impact. Discrepancies between genotypic and phenotypic resistance occur, highlighting the complexity of the mechanisms of antimicrobial resistance (AMR) and the importance of investigating resistance at both levels [7]. Indeed, previous studies have shown that combining phenotypic and genotypic characterization improves the prediction of AMR resistance patterns and informs more accurate strategies for surveillance and control [8].

Numerous studies on ESBL‐Ec in the European Union have focused on specific species or geographic areas [9–12]. In Switzerland, efforts have focused on swine, calves, and poultry, as highlighted in the Swiss Antibiotic Resistance Report [13], but data on indicator *E. coli* in wildlife are scarce. Complementing these ongoing surveillance programs is important as wildlife differ significantly in terms of antimicrobial selection pressure, habitats, ecological niches, and interactions with humans, representing two distinct potential reservoirs of ESBL‐Ec.

In this study, we aimed to (a) estimate the prevalence of ESBL‐producing *E. coli* in dairy cattle and wildlife, (b) characterize phenotypic coresistance across antimicrobial classes, and (c) integrate whole‐genome sequencing (WGS) for genotypic resistance characterization. This study reports the prevalence, AMR epidemiology, resistance profiles, and clonal dynamics of ESBL‐Ec in Swiss dairy cattle and wildlife, offering new data to refine surveillance strategies and inform targeted mitigation efforts.

## 2. Material and Methods

### 2.1. Sample Collection

Samples were retrieved from two field studies previously conducted by our research group investigating AMR in Swiss dairy cows and wildlife between 2021 and 2023 [14, 15]. Target populations included nationwide dairy cattle and free‐roaming wild mammals and birds from Switzerland and the Principality of Liechtenstein. Both live and recently hunted or deceased animals were sampled. Rectal or cloacal mucosa swabs were collected using sterile cotton swabs (Copan Transystem, Brescia, Italy, and Meus S.r.l., Padova, Italy) inserted into the rectum or cloaca and placed immediately into a transport medium suitable for Enterobacteriaceae. For small migratory birds sampled during ringing activities, fecal samples were collected opportunistically from droppings deposited on sterile surfaces during transport and handling. Birds were transported individually in cloth bags lined with a sterile inner layer, and the ringing bench was covered with a sterile disposable liner before handling each bird. When droppings were deposited on either sterile surface, the fecal material was collected immediately for analysis. Samples were transported to accredited laboratories either via a courier on the day of collection or by overnight mail. Livestock samples were collected by the study team, whereas wildlife samples were mostly obtained through collaboration with wildlife guardians, hunters, and ornithologists. Wildlife sampling was opportunistic and availability‐based and was not designed to be proportional to species abundance or to yield population‐representative prevalence estimates across all wild animals. Ethical approvals for all sampling procedures were obtained (permits BE76/2021, P38, and BE61/2020/32505).

### 2.2. Isolation and Identification of ESBL‐Ec

Rectal swabs were streaked onto CHROMagar ESBL chromogenic media (CHROMagar ESBL, Paris, France) without prior dilution the day after swab sample collection and incubated at 37°C for 24 h. Up to three dark pink to reddish colonies per plate, indicative of *E. coli*, were selected and confirmed by matrix‐assisted laser desorption/ionization time‐of‐flight mass spectrometry (MALDI‐TOF M, Bremen, Germany). Animals were considered ESBL‐Ec‐positive if at least one colony from the three was confirmed as *E. coli* by MALDI‐TOF MS. The number of ESBL‐Ec‐positive animals was subsequently recorded. Additionally, 16 ESBL‐Ec isolates from a parallel Swiss dairy cattle study conducted during the same sampling period (2021–2023) were included for WGS and phenotypic susceptibility testing [14]. These isolates originated from rectal swabs of dairy cattle sampled across multiple cantons during winter housing following recent parenteral antimicrobial treatment and were initially isolated using selective culture media and MALDI‐TOF MS‐based identification. These isolates were included solely for genomic and phenotypic characterization to provide additional genomic context for cattle‐associated ESBL‐Ec and were not included in the prevalence calculations reported in the present study. Recruitment and sample collection took place between October 2021 and January 2023. Prior to inclusion in the present study, all previously collected isolates were recultured and rescreened on CHROMagar ESBL using the same protocol as that applied to newly collected samples to ensure methodological consistency. Colonies growing on CHROMagar ESBL were considered putative ESBL‐Ec. ESBL production was subsequently confirmed by antimicrobial susceptibility testing and detection of ESBL genes from WGS.

### 2.3. Phenotypic Susceptibility Testing for Antimicrobials

Antimicrobial susceptibility testing followed the Clinical and Laboratory Standards Institute (CLSI) disk diffusion guidelines [16], using 16 drugs from 10 antimicrobial classes. Antimicrobial classes tested were (μg/disc): tetracyclines (tetracycline [30]), sulfonamides (trimethoprim‐sulfamethoxazole [1.25/23.75]), penicillins (ampicillin [10], amoxicillin‐clavulanic acid [20/10]), cephalosporins (cefazolin [30], cefotaxime [30], cefepime [30], ceftazidime [30]), phenicols (chloramphenicol [30]), aminoglycosides (gentamicin [10], streptomycin [10]), fluoroquinolones (ciprofloxacin [5], nalidixic acid [30]), glycylcyclines (tigecycline [15]), macrolides (azithromycin [15]), and carbapenems (meropenem [10]) (Bio Rad, Marnes‐la‐Coquette, France). *E. coli* ATCC 25,922 was used as the control strain (Remel Inc., Lenexa, USA). Each isolate was cultivated on brain‐heart infusion (BHI) agar plates with 5% sheep blood (Bio Rad, Marnes‐la‐Coquette, France), incubated at 35 ± 2°C overnight, and standardized to 0.5 McFarland turbidity. The bacterial suspension was spread onto Mueller‐Hinton (MH) agar plates using a sterile cotton swab. Afterwards, disks containing antimicrobial drugs were positioned on the inoculated MH agar plates using a disk dispenser (Bio Rad, Marnes‐la‐Coquette, France). Plates were incubated aerobically at 35 ± 2°C for 16–18 h, and inhibition zones were measured using a calibrated ruler. Interpretive criteria by CLSI were used to classify isolates as susceptible (S), intermediate (I), or resistant (R) [16]. For tigecycline, interpretive criteria of the European Committee on Antimicrobial Susceptibility Testing (EUCAST) were applied, as no CLSI breakpoints were available [17]. Intermediate isolates were considered susceptible. Multidrug resistance (MDR) was defined as resistance to at least one drug from three or more antimicrobial classes [18].

### 2.4. DNA Extraction and WGS

DNA was extracted using the DNeasy Blood and Tissue kit (Qiagen, Hilden, Germany) following the manufacturer’s instructions, starting from 1 mL of enriched liquid culture in Luria Broth (AppliChem GmbH, Darmstadt, Germany) and eluting in 100 μL buffer AE. WGS was performed using the LITE Library Prep method and Illumina sequencing on a NextSeq 1000 P2 flow cell (300 bp paired‐end) at the Earlham Institute, Norwich, UK [19].

### 2.5. Bioinformatic Analyses and Isolates Characterization

Illumina reads were trimmed using Trimmomatic v0.35 in paired‐end mode, with Illumina Nextera adapters removed (parameters: 2:30:10:1) [20]. Quality trimming employed a 4 bp sliding window (quality ≥20), and reads <36 bp were discarded. Read quality pre‐ and posttrimming was assessed with FastQC v0.11.4 [21]. Contamination screening was performed with FastQ Screen v0.15.3 [22] mapping reads to seven reference genomes from NCBI: *Acinetobacter baumannii* (ASM863263v1), *Citrobacter freundii* (ASM381234v1), *Escherichia coli* (MG1655), *Enterobacter roggenkampii* (ASM172980v1), *Klebsiella pneumoniae* (ASM24018v2), *Pseudomonas aeruginosa* (ASM676v1), and *Proteus mirabilis* (ASM6996v1). These species were chosen due to their potential to grow on CHROMagar ESBL plates and coculture with *E. coli*. Reads were assembled de novo using SPAdes v3.15.9 [23] with error correction disabled and the “careful” option enabled. Genome annotation was conducted with Bakta v1.9.1 [24]. Sequence types (STs) were identified via the Achtman MLST scheme using MLST v2.23.0 (https://github.com/tseemann/mlst), and phylogenetic groups were assigned using EzClermont v0.6.3 [25]. Antibiotic resistance genes (ARGs) were detected with Abricate v1.0.1 [26], blasting against the CARD database (accessed 4th November 2023) [27]. ARGs were classified by antibiotic class based on the CARD annotations.

### 2.6. Core‐Genome Analyses and Phylogenetic Reconstruction

The pangenome was generated using Roary v3.13.0 [28] to assess genetic diversity, with core genes (present in ≥99% of isolates) aligned by MAFFT for subsequent analyses. Pairwise SNP differences were calculated using an in‐house Python script (https://github.com/banklab/ESBL) and are summarized in Table S1. Because core‐genome SNP distances do not capture variation in accessory genomic regions where AMR determinants often reside, multiple isolates from the same animal were retained for phenotypic and genotypic analyses even when core‐genome SNP differences were zero. A cutoff of 20 SNPs was used to define clonal isolates among different individuals (Table S2). This value was chosen based on the empirical distribution of pairwise SNP distances in our dataset, where 20 SNPs marked the separation between closely related and more distant isolates and is consistent with commonly used thresholds for *E. coli* in genomic epidemiology studies. Plasmid content was identified via PlasmidFinder‐2.0 using the Enterobacteriales database, with a threshold of ≥95% identity and ≥ 60% coverage, and the raw paired‐end fastq reads as input [29].

Phylogenetic analysis was performed using IQ‐TREE 2 [30] on 55 sequences, including 54 ESBL‐Ec isolates and one outgroup (*Escherichia albertii*, NCBI: ASM1690475v2). The input alignment (2’212’064 sites) contained 86.74% constant sites, 149’328 parsimony‐informative sites, and 58’407 distinct site patterns. The best‐fit substitution model (GTR + F+R5) was selected using ModelFinder [31] based on the Bayesian Information Criterion. Maximum likelihood was used to infer tree topology, with branch support assessed via Ultrafast Bootstrap Approximation (UFBoot2) [32] with 1’000 replicates. The tree was visualized and annotated in iTOL [33].

### 2.7. Statistical Analyses

The prevalence of putative ESBL‐Ec was calculated separately for dairy cows and wildlife as the proportion of ESBL‐positive animals among all sampled animals. Wildlife prevalence was further stratified by major taxonomic groups, namely, birds and mammals. For each prevalence estimate, two‐sided 95% confidence intervals (CIs) were calculated using the Wilson Score binomial method without continuity correction, which yields reliable interval coverage for low‐prevalence data and small sample sizes [34]. Analyses were conducted in R v.4.5.1 and RStudio v.2025.09.0.

## 3. Results

### 3.1. Study Populations and Prevalence of ESBL‐Ec

**Figure 1:**
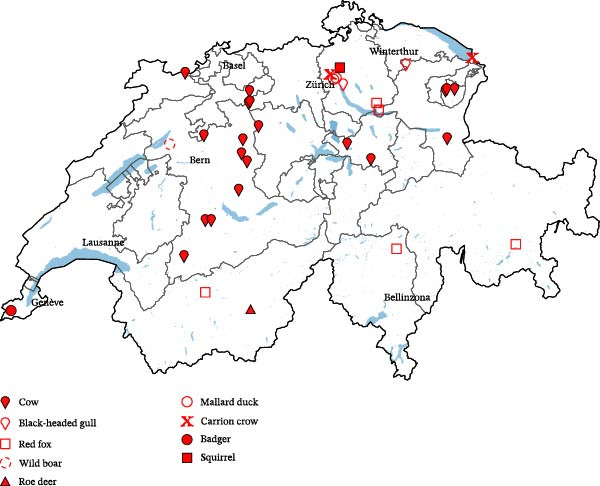
Map of Switzerland showing the geographic distribution of sampled livestock and free‐roaming wildlife harboring extended‐spectrum β‐lactamase (ESBL)‐producing *Escherichia coli* collected between 2021 and 2023. Sampling locations are indicated by symbols corresponding to animal species, as shown in the legend (cow, black‐headed gull, red fox, wild boar, roe deer, mallard duck, carrion crow, badger, and squirrel). Canton borders and major lakes are displayed for geographic reference. The map was generated in R v2025.09.0 + 387 and further modified in Inkscape v1.4.2. ESBL‐*E. coli* isolates from one badger, one squirrel, one carrion crow (Zurich), and one red fox (Valais) failed whole‐genome sequencing and were excluded from genotypic‐phenotypic comparisons and the phylogenetic tree.

A total of 300 fecal samples from wildlife and 475 rectal samples from dairy cows collected from 131 farms were streaked to isolate putative ESBL‐Ec. The sampled wildlife population comprised 23 bird species (*n* = 99%, 33.3%) and 15 mammal species (*n* = 200%, 66.6%), along with one unidentified animal. The overall prevalence of putative ESBL‐Ec was 0.6% (95% CI 0.2%–1.8%) in dairy cows (3 positive animals) and 5% (95% CI 3.1%–8.1%) in wildlife (15 positive animals, Table 1). When stratified by wildlife groups, putative ESBL‐Ec was detected in six bird individuals (6.1%, 95% CI 2.8%–12.6%) and 9 mammal individuals (4.5%, 95% CI 2.4%–8.3%), indicating a slightly higher prevalence in birds than in mammals (Table 1). Red foxes, European roe deer, wild boars, and cormorants were the most frequently represented wildlife species (Table S3). Wildlife animals positive for putative ESBL‐Ec included red foxes (*n* = 5; 7.1%, 95% CI 3.1%–15.7%), black‐headed gulls (*n* = 2; 40.0%, 95% CI 11.8%–76.9%), mallard ducks (*n* = 2; 13.3%, 95% CI 3.7%–37.9%), carrion crows (*n* = 2; 13.3%, 95% CI 3.7%–37.9%), and single positives in wild boar (*n* = 1; 4.3%, 95% CI 0.8%–21.0%), European roe deer (*n* = 1; 1.9%, 95% CI 0.3%–9.8%), European badger (*n* = 1; 9.1%, 95% CI 1.6%–37.7%), and squirrel (*n* = 1; 25%, 95% CI 4.6%–69.9%) (Table S3). The carrion crow sample was imported from Austria prior to the shipment to the laboratory, and the cantonal authorities confirmed that no additional permits were required. The prevalence estimates reported here are based exclusively on the animals screened in this study. The 16 additional ESBL‐Ec isolates collected during the same period in a parallel study were included only for the phenotypic and genotypic characterization analyses presented below and were not used in the prevalence calculations. Sampling locations for dairy farms and wildlife, along with ESBL‐Ec locations, including those retrieved from a previous study, are shown in Figure 1.

**Table 1:** Study populations, putative ESBL‐*E. coli* prevalence, isolate yield, and 95% confidence intervals.

Population	*N* animals	ESBL positive animals	Prevalence (%)	95% CI (%)	ESBL‐Ec isolates retrieved^a^	WGS successful	Animals contributing to WGS
Dairy cows	475	3	0.6	0.2–1.8	26^b^	25^c^	19
Wildlife (total)	300	15	5	3.1–8.1	45	29^c^	11
Birds	99	6	6.1	2.8–12.6	18	13	5
Mammals	200	9	4.5	2.4–8.3	27	16	6
Unidentified wildlife	1	0	NA	NA	NA	NA	NA

*Note: “N* animals” denotes the number of sampled animals per group; “ESBL positive” animals the number of animals from which ESBL‐producing *E. coli* was isolated; “Prevalence (%)” is ESBL positive animals over total number of animals. “95% confidence intervals (CI)” were calculated using the Wilson binomial method. “Animals contributing to WGS” indicates the number of ESBL‐positive animals from which successfully sequenced isolates were obtained.

^a^Up to three ESBL‐*E. coli* isolates were retrieved per positive animal.

^b^Dairy cow isolates include 17 from a parallel Swiss study and 9 from the present sampling design.

^c^One dairy cow isolate and 16 wildlife isolates had no/poor WGS and were excluded from genotype‐phenotype comparisons. A total of 54 ESBL‐*E. coli* genomes were successfully sequenced (25 dairy cows, 29 wildlife).

### 3.2. AMR Phenotypes

**Figure 2:**
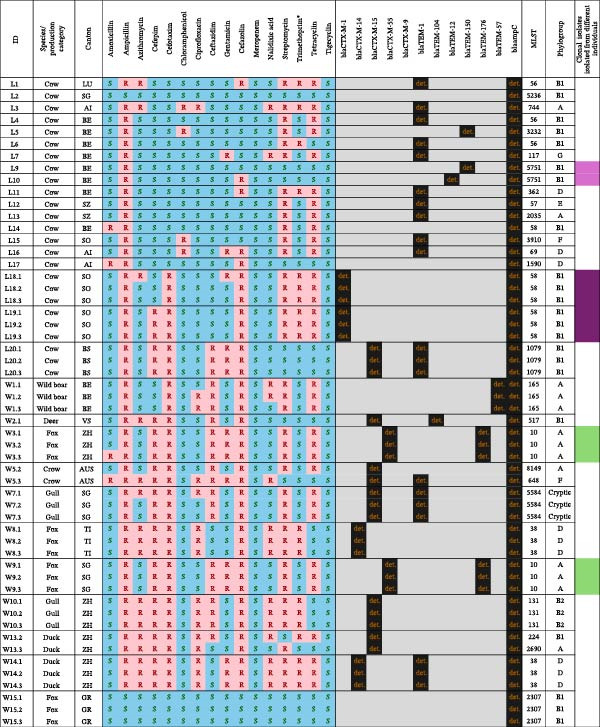
Phenotypic antimicrobial resistance profile, occurrence of genes conferring beta‐lactam resistance, and multilocus sequence types (MLST) in 54 *Escherichia coli* isolates from Swiss livestock and wildlife, 2021–2023. Isolates L1‐L17 originate from a parallel study conducted during the same period and were included to expand the phenotypic‐genotypic comparison. All other isolates originate from the prevalence study described in this manuscript. Clonal isolates obtained from different individuals are marked with colored bars in the far‐right column. Susceptible (S) and resistant (R) phenotypes are shown in green and red, respectively. Dark green (on green) and dark red (on red) are used to visually emphasize the phenotypic classification within each antimicrobial category. L, livestock; W, wildlife; S, susceptible; R, resistant; det. = gene detected,  ^∗^sulfamethoxazole,  ^∗∗^from different animals; species: duck = mallard duck, gull = black‐headed gull, crow = carrion crow; cantons: Appenzell Inner Rhodes (AS), Bern (BE), Basel‐Stadt (BS), Grisons (GR), Schwyz (SZ), St. Gallen (SG), Solothurn (SO), Ticino (TI), Valais (VS), AUS = Fussach/Austria.

In total, 54 putative ESBL‐Ec isolates (25 from dairy cows and 29 from wildlife) with successful WGS were included in phenotypic antimicrobial susceptibility testing and genotypic analyses. Of the 54 putative ESBL‐Ec isolates recovered on CHROMagar ESBL, 35 were confirmed ESBL producers based on cefotaxime resistance and/or presence of an ESBL gene (Figure 2). Phenotyping revealed that 93% (*n* = 50) of the putative ESBL‐Ec isolates exhibited resistance to ampicillin, 7% (*n* = 4) to amoxicillin, 74% (*n* = 40) to cefazolin, 65% (*n* = 35) to the 3rd‐generation cephalosporin cefotaxime, 41% (*n* = 22) to ceftazidime, and 54% (*n* = 29) to the 4th‐generation cephalosporin cefepime (Figure 1). Resistance to other antimicrobial agents included streptomycin (76%, *n* = 41), tetracycline (63%, *n* = 34), trimethoprim‐sulfamethoxazole (37%, *n* = 20), ciprofloxacin and nalidixic acid (30%, *n* = 16), gentamycin (30%, *n* = 16), and chloramphenicol (7%, *n* = 4) resistance. No phenotypic resistance to tigecycline or meropenem was observed. Overall, 89% of putative ESBL‐Ec isolates exhibited a MDR phenotype.

### 3.3. AMR Genotypes

**Figure 3:**
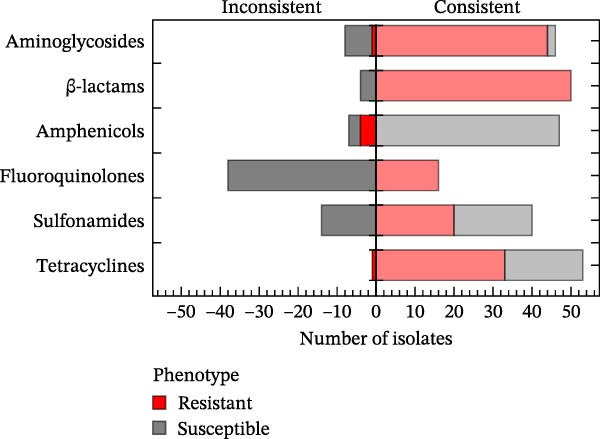
Concordance between phenotypic resistance and antimicrobial resistance gene (ARG) detection across six antibiotic classes. Bars represent the number of isolates with consistent (right of the center line) or inconsistent (left of the center line) results. Consistent results include resistant isolates with the corresponding ARGs (red) and susceptible isolates without the corresponding ARGs (gray). Inconsistent results include resistant isolates without ARGs (red) and susceptible isolates with ARGs (gray).

Among the sequenced putative ESBL‐Ec, 89% (*n* = 48) harbored at least one beta‐lactamase gene (Figure 2). The most frequent gene was bla_TEM-1_, detected in 37% (*n* = 20) of putative ESBL‐Ec, followed by bla_CTX-M−15_, detected in 26% (*n* = 14) of putative ESBL‐Ec. Overall, four different bla_CTX-M_ variants and six different bla_TEM_ variants were detected (Figure 2). In decreasing order, the most frequent bla_CTX_ genes were bla_CTX-M−15_ (25.9%, *n* = 14), bla_CTX-M−55_ (11.1%, *n* = 6), bla_CTX-M−14_ (11.1%, *n* = 6), and bla_CTX-M−1_ (11.1%, *n* = 6). Among bla_TEM_ variants, bla_TEM-1_ occurred most frequently (37.0%, *n* = 20), followed by bla_TEM-176_ (11.1%, *n* = 6), bla_TEM-150_ (3.7%, *n* = 2), and bla_TEM-104/-12/-57_ (1.9%, *n* = 1 each). Additionally, 11 genes conferring resistance to aminoglycosides, 5 to fluoroquinolones, 3 to sulfonamides/diaminopyrimidines, 4 to tetracycline, 6 to peptide antimicrobials, 2 to macrolides, and 1 to lincosamides were detected (Table S4). The total phenotypic and genotypic concordance (i.e., isolates either resistant with ARGs or susceptible without ARGs) was highest for tetracyclines (98%, *n* = 53), followed by beta‐lactams (93%, *n* = 50), amphenicols (87%, *n* = 47), aminoglycosides (85%, *n* = 46), sulfonamides/diaminopyrimidines (70%, *n* = 38), and fluoroquinolones (30%, *n* = 16) (Figure 3 and Table 2). Notably, it was more frequent for a strain to be phenotypically susceptible but carry a resistance gene than to be phenotypically resistant without a corresponding ARG, except for tetracyclines and amphenicols (Figure 3 and Table 2).

**Table 2:** Phenotypic and genotypic concordance for antimicrobial resistance among ESBL‐producing *E. coli* isolates, grouped by antimicrobial class.

Antimicrobial class	Resistant without ARG (%, *n*)	Susceptible with ARG (%, *n*)	Resistant with ARG (%, *n*)	Susceptible without ARG (%, *n*)	Resistant with ARG or susceptible without ARG (%, *n*)
Aminoglycosides	2%, 1	13%, 7	81%, 44	4%, 2	85%, 46
β‐lactams	0%, 0	7%, 4	93%, 50	0%, 0	93%, 50
Amphenicols	7%, 4	6%, 3	0%, 0	87%, 47	87%, 47
Fluoroquinolones	0%, 0	70%, 38	30%, 16	0%, 0	30%, 16
Sulfonamides	0%, 0	26%, 14	37%, 20	37%, 20	74%, 40
Tetracyclines	2%, 1	0%, 0	61%, 33	37%, 20	98%, 53

*Note:* The table presents the percentage and number of isolates classified into five categories: “Resistant without ARG” (phenotypic resistance in the absence of known resistance genes), “Susceptible with ARG” (presence of resistance genes despite phenotypic susceptibility), “Resistant with ARG” (phenotypic resistance with corresponding resistance genes), “Susceptible without ARG” (phenotypic susceptibility with no detected resistance genes), and “Resistant with ARG or Susceptible without ARG” (combined concordance category, representing isolates with matching phenotypic and genotypic resistance or susceptibility). Percentages (%) and absolute numbers (*n*) are provided for each category.

### 3.4. Phlyogenetic Distribution of Putative ESBL‐Ec and Clonal Relationship

**Figure 4:**
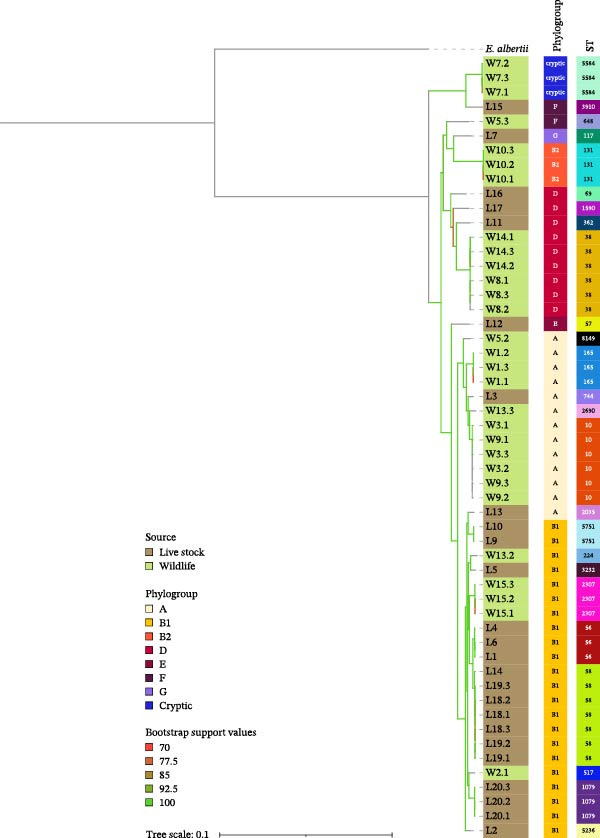
Phylogenetic tree of 54 extended‐spectrum beta‐lactamase (ESBL)‐producing *Escherichia coli* isolates from Swiss livestock and free‐roaming wildlife, collected between 2021 and 2023, along with one outgroup (*Escherichia albertii*). The tree illustrates the distribution of phylogroups, sequence types (STs), and sources of collection. Sources are color‐coded: brown for livestock and green for wildlife. Phylogroups (A, B1, B2, D, E, F, G, and cryptic) are highlighted with distinct colors. Sequence types (STs) are annotated, with notable STs explicitly labeled. The tree was constructed using IQ‐TREE with automatic model selection, and branch lengths represent substitutions per site, as indicated by the scale bar. Bootstrap support values, ranging from 70% (red) to 100% (green), are represented by the color gradient of the branches, with no values below 70% observed.

The maximum‐likelihood phylogenetic tree constructed from a core gene alignment of 54 putative ESBL‐Ec isolates revealed diverse clustering across eight phylogroups, with the most frequent being B1 (39%, *n* = 21), followed by A (24%, *n* = 13), D (15%, *n* = 8), and B2, E, and cryptic (6%, *n* = 3 each) (Figure 4). Phylogroup B2 was exclusively observed in gull isolates and corresponded to ST131, a lineage known for its multidrug‐resistant phenotype. Among the isolates, 28 unique STs were identified, with the most frequent being ST58 (13%, *n* = 7), followed by ST10 and ST38 (11%, *n* = 6 each) and ST56, ST131, and ST165 (6%, *n* = 3 each). Wildlife isolates displayed diversity in STs, with ST38 observed in foxes and ducks and ST10 in foxes, ST131 in gulls, and ST165 in wild boars. ST165 was exclusively detected in wild boars, and ST131 was restricted to gulls. Livestock isolates clustered more narrowly, with ST58 being the predominant ST and being exclusively found in cattle.

The phylogenetic tree shows a structured distribution of isolates by source with some degree of intermixing (Figure 4). Wildlife‐derived isolates (green) predominantly cluster in the upper part of the tree, primarily within phylogroups A, D, and cryptic. In contrast, livestock‐derived isolates (brown) are more concentrated in the lower clades, particularly within phylogroup B1. Notable exceptions include a wildlife‐derived isolate (W2.1, deer) clustering within a primarily livestock‐associated clade in phylogroup B1. Similarly, livestock‐derived isolates L3 and L13 in phylogroup A cluster within a clade that is predominantly associated with wildlife, reflecting overlapping genetic diversity between the two compartments. Plasmid replicon typing revealed diverse plasmid content among ESBL‐Ec isolates from both livestock and wildlife, with IncFIB and IncI1‐I detected across host compartments and some isolates carrying multiple replicon types (Table S5).

Genetic similarity indicated genetic clusters in both livestock and wildlife, resulting in a bimodal distribution of SNP differences between isolates (Table S2). Livestock isolates exhibited either few SNP differences or >400 (mean: 40,178 SNPs), and wildlife isolates had either <20 SNP differences or >37 (mean: 46,900 SNPs) (Table S1). Clonal relationships were identified among several isolates from different individuals. For example, livestock isolates L9 and L10 had only 2 SNPs differences, whereas isolates L18.1‐3 and L19.1‐3 differed by 0–8 SNPs (Table S1). Similarly, wildlife isolates W3.1‐3 and W9.1‐3, all from red foxes in Zurich, demonstrated high degrees of genetic similarity: W3.1 and W9.1 were identical (0 SNPs), and also comparisons between W3.1 and W9.2 (19 SNPs) and W3.1 and W9.3 (16 SNPs) indicated close relatedness between the isolates (Table S2). In contrast, SNP differences between W3.3 and W9 isolates were larger (105–123 SNPs), suggesting greater genetic divergence.

## 4. Discussion

This study investigated the prevalence, phenotypic and genotypic AMR profiles, and phylogenetic distribution of ESBL‐Ec in dairy cattle and wildlife in Switzerland from samples collected between 2021 and 2023. The prevalence of putative ESBL‐Ec was 0.6% (95% CI 0.2–1.8%) in dairy cattle and 5% (95% CI 3.1–8.1%) in wildlife, with isolates detected across multiple species. Concordance between phenotypic and genotypic resistance was highest for tetracyclines (98%, *n* = 53) and beta‐lactams (93%, *n* = 50), but discordance occurred when isolates were phenotypically susceptible despite harboring resistance genes or vice versa, especially for fluoroquinolones and sulfonamides. WGS revealed a high genetic diversity between the isolates. Twenty‐eight unique STs were identified, with phylogroup B1 being the most frequent (39%, *n* = 21). Close genetic relationships were observed within and between animals, highlighting potential transmission dynamics. The presence of multidrug‐resistant lineages, such as ST131 in gulls and ST38 in foxes and ducks, underscores the public health threat posed by bacteria that reside in environmental reservoirs.

The prevalence of putative ESBL‐Ec in wildlife (5%) aligns with studies from Switzerland, Europe, and overseas, where percentages ranged from 1.1% to 6.8% [5, 35, 36]. When stratified by wildlife groups, we observed a higher prevalence in birds (6.1%) than in mammals (4.5%). This pattern is consistent with previous studies reporting higher ESBL‐Ec carriage in birds, including a Swiss study detecting ESBL‐Ec in 6.8% of wild birds [5] and a study from Brazil reporting 2.4% prevalence in avian species [35]. In contrast, lower prevalence rates have been reported among wild mammals, including 1.2% in wild boars and 1.1% in wild ruminants, in Germany [36]. Higher ESBL‐Ec prevalence in birds may reflect differences in ecology and behavior, such as broader spatial movement, frequent use of aquatic or anthropogenically impacted habitats, and increased exposure to environmental contamination from wastewater, agricultural runoff, or land‐applied manure. Higher rates (52%–67%) previously reported from peridomestic wildlife highlight regional and ecological variability of ESBL‐Ec prevalence [37, 38]. Based on comparison with a Swiss wildlife report from 2011, ESBL‐Ec prevalence in wild ungulates may have remained stable, as our study detected one positive isolate (corresponding to 1.4%) compared to 0.4% reported previously [39]. The detection of ESBL‐Ec in wildlife is expected given the frequent use of antimicrobials in livestock, potential direct and/or indirect contact on pastures, and farm effluents, which are dispersed onto land without prior treatment, potentially contributing to environmental contamination. Notably, our study design did not allow us to determine whether wildlife serves as a transient host or a long‐term reservoir for ESBL‐Ec. Because several wildlife species were represented by small sample sizes, species‐specific prevalence estimates should be interpreted with caution. The detection of ESBL‐Ec in wildlife likely reflects environmental exposure to anthropogenic sources, such as agricultural environments or wastewater contamination, rather than sustained wildlife‐specific reservoirs.

We found a prevalence of putative ESBL‐Ec of 0.6% (95% CI 0.2–1.8%) in livestock, which is much lower than the numbers reported from overseas [37, 40, 41] and from Switzerland. For example, studies from Madagascar and the United States reported substantially higher prevalence in cattle, but these relied on a pre‐enrichment step prior to culturing, which increases sensitivity for detecting low‐abundance ESBL‐Ec [37, 41]. In addition, one U.S. study reporting an 8.5% prevalence quantified cefotaxime‐resistant bacteria without restriction to *E. coli*, limiting direct comparability with our culture‐based ESBL‐Ec approach [40]. In Switzerland, a previous study found a 13.7% prevalence in cattle feces, though this was based on a smaller sample size and used an enrichment step before culturing, which increases detection sensitivity [42]. In contrast, our study applied direct plating without enrichment, which likely reduced sensitivity for detecting ESBL‐Ec present at low abundance. Accordingly, the low prevalence observed here should be interpreted as a conservative estimate of ESBL‐Ec carriage in Swiss dairy cattle rather than an absence of colonization. Additionally, routine surveillance of veal calves at slaughterhouses in Switzerland has reported an ESBL/ampC‐Ec prevalence of around 30% [13]. Because these estimates are based on postmortem cecal contents rather than rectal swabs and given additional differences in sample matrix (cecal content vs. feces vs. rectal swabs), enrichment strategy, animal age, and sampling context (surveillance vs. research), direct quantitative comparison is limited and likely contributes to the lower prevalence observed in our study.

We found that MDR was a prominent feature among the putative ESBL‐Ec isolates, with 89% exhibiting resistance to at least one antimicrobial drug from three or more distinct classes. Besides beta‐lactams, phenotypic resistance was most frequent for aminoglycosides, tetracyclines, and sulfonamides, whereas no resistance was detected against tigecycline or meropenem, indicating that these clinically relevant antimicrobials remain effective. The high MDR prevalence in both livestock and wildlife raises concerns about the potential transmission of multiple resistance types to other pathogenic bacteria via horizontal gene transfer (HGT).

Genetic analysis revealed a diverse range of AMR genes, with bla_TEM-1_ (37%, *n* = 20) and bla_CTX-M-15_ (26%, *n* = 14) as the most prevalent beta‐lactamases. Additional resistance determinants for aminoglycosides, fluoroquinolones, sulfonamides/diaminopyrimidines, tetracyclines, peptide antimicrobials, macrolides, and lincosamides were detected. The co‐occurrence of multiple resistance genes across unrelated antimicrobial classes may reflect HGT via mobile genetic elements, though independent acquisition cannot be ruled out. The detection of IncFIB and Inc11‐I plasmids in isolates from dairy cattle barns, wild mammals, and birds suggests that these plasmid types may play a role in the dissemination of resistance traits across different environments. Our findings call for in‐depth studies of the transmission routes of bacteria and plasmids, particularly given the spatiotemporal variability associated with the sampled free‐roaming wildlife. Future research should assess whether the spread of AMR via bacteria and/or via plasmids can be mitigated and identify effective intervention strategies.

We found a notable level of non‐concordance between phenotypic and genotypic resistance. Isolates were more frequently phenotypically susceptible despite carrying resistance genes than phenotypically resistant without the corresponding genes. The susceptibility of carriers of resistance genes can potentially be attributed to gene expression variability, differences in promoter activity, or the presence of nonfunctional resistance genes [43]. In particular, fluoroquinolone resistance is often driven by point mutations in chromosomal target genes such as *gyrA* and *parC*, which were not systematically screened in this study [44]. The absence of such mutations likely explains why isolates carrying plasmid‐mediated fluoroquinolone resistance genes remained phenotypically susceptible as these determinants alone often confer only low‐level resistance [45]. Conversely, phenotypic resistance without a detectable gene might indicate alternative and potentially unknown resistance mechanisms, such as via efflux pumps or mutations in target sites [46]. Furthermore, CHROMagar ESBL recovered a small proportion of non‐ESBL‐Ec, consistent with its role as a screening rather than a confirmatory medium, highlighting the importance of phenotypic or genotypic confirmation. Together, these findings underline that gene‐based screening approaches, while informative, incompletely capture the phenotypic resistance landscape, particularly for resistance mechanisms driven by chromosomal variation or regulatory contexts.

Spatial proximity was an important factor related to ESBL‐Ec genetic similarity. In wildlife, fox isolates W3.1‐3 and W9.1‐3, collected 2 km apart at the Zurich‐St. Gallen border over a 5‐week interval, were found to be completely clonal, including their plasmids, suggesting local persistence and potential transmission. In livestock, clonal isolates were frequently detected within the same barns. Dairy cows from tie‐stall barns, including isolates L9 and L10 from cows tied at opposite ends of a barn and L18.1‐3 and L19.1‐3 from another barn under similar conditions, carried genetically similar isolates with less than a 10 SNP difference. The close genetic relatedness of the isolates suggests that confined spaces facilitate the clonal spread of ESBL‐Ec independent of antibiotic treatment in livestock [47]. Future studies are needed to quantify the duration for which ESBL‐Ec persists in barns after cessation of antibiotic treatment. At a larger scale, phylogenetic analysis revealed some intermixing between livestock and wildlife isolates, with wildlife isolate W2.1 clustering in phylogroup B1, predominantly associated with livestock, and livestock isolates L3 and L13 clustering in phylogroup A, commonly linked to wildlife. However, no ST was shared between livestock and wildlife, suggesting ecological and behavioral barriers limiting direct transmission, although the limited sample size may also have reduced our ability to detect overlap. The observed phylogenetic proximity may also reflect parallel acquisition through shared environmental reservoirs, such as surface waters or landscapes impacted by manure application and agricultural run‐off, which could transiently expose wildlife to livestock‐associated lineages without leading to long‐term establishment [48]. The predominance of phylogroup B1 and the exclusive detection of ST58 in cattle indicate livestock as potential reservoirs for specific lineages, whereas ST38 detection in foxes and ducks highlights its adaptability and potential spread across wildlife species.

In Switzerland, *E. coli* ST131 has been repeatedly detected in water and wastewater [6]. ST131 has spread globally since the early 2000 s and is a major cause of severe urinary tract infections, bloodstream infections, and sepsis in humans [49]. ST131 is a clinically dominant high‐risk clone that combines extensive AMR, frequently including ESBLs, with enhanced virulence, contributing to treatment failure and recurrent infections in both hospital and community settings [50]. Its association with water aquatic environments is well‐documented [51–53]. Our detection of ST131 in one sample from a gull, a species adapted to aquatic habitats, aligns with reports of ST131 from fish and highlights its environmental persistence and potential spread across a broad host range. ST38, while less dominant in clinical settings than ST131, is also recognized as a globally distributed multidrug‐resistant lineage of clinical relevance [53]. It is frequently associated with ESBL production and has been implicated in community‐acquired infections while exhibiting strong ecological versatility [54]. ST38 has been previously detected in various Swiss environments, including surface water, fish, vegetables, poultry, poultry meat, and healthy humans [55–57]. Our detection of ESBL‐Ec ST38 in a duck north of the Alps (phylogroup D) and in a fox from Ticino in southern Switzerland (phylogroups D and E) supports its adaptability to a broad host range and suggests widespread circulation in wildlife on both sides of the geographical barrier posed by the Alpine range.

This study has several limitations. First, the low ESBL‐Ec prevalence observed in livestock may partly reflect the use of direct plating without a pre‐enrichment step, which likely reduced the sensitivity for detecting low‐abundance ESBL‐Ec and may have led to an underestimation of the true prevalence in the livestock population. Second, disk diffusion testing, while standardized, lacks MIC values, limiting the insight into resistance levels. Third, WGS, though informative, does not capture all mobile genetic elements or plasmids within one ESBL‐Ec isolate. Additionally, although isolates with zero core‐genome SNP differences from the same host were retained to capture within‐host phenotypic diversity, core‐genome analyses do not fully resolve variation in the accessory genome, where many AMR determinants reside; thus, some closely related isolates classified as clonal at the core‐genome level may still differ in the resistance phenotype. In addition, lineage‐specific core‐genome SNP analyses within individual MLST/cgMLST types were not performed. Such approaches can provide higher phylogenetic resolution among closely related isolates but require larger numbers of genomes per lineage than available here as most STs were represented by few isolates. Furthermore, the cross‐sectional design prevents assessing temporal persistence. Longitudinal studies are needed to evaluate clonal stability and the role of livestock and wildlife in sustaining resistance. Finally, wildlife sampling was not proportional across species; therefore, prevalence estimates are most appropriately interpreted at the wildlife group (birds vs. mammals), whereas the overall wildlife prevalence should be viewed as descriptive of the sampled population rather than extrapolated to all wildlife.

In conclusion, this study confirms the presence of ESBL‐Ec in both livestock and wildlife across Swiss environments (2021–2023), with evidence of clonal dissemination within each host group. Between host groups, however, ESBL‐Ec showed distinct evolutionary trajectories and no shared STs. The occasionally observed phylogenetic intermixing may reflect indirect connectivity via shared environmental reservoirs or cross‐host transmission. The observed high prevalence of MDR is particularly concerning because it indicates that it is most common for multiple resistance types to be maintained and transmitted jointly. From a One Health perspective, our findings underscore the need for integrated intervention strategies that extend beyond clinical and agricultural settings. Such strategies include coordinated AMR surveillance across humans, livestock, wildlife, and environmental compartments; improved manure and agricultural run‐off management to limit environmental dissemination; and targeted monitoring of wildlife species associated with aquatic and anthropogenically influenced habitats.

## Author Contributions

**Sheena Conforti**: conceptualization, data curation, formal analysis, investigation, methodology, project administration, software, validation, visualization, writing – original draft, writing – review and editing. **Adamandia Kapopoulou**: data curation, formal analysis, investigation, methodology, software, writing – review and editing. **Claudia Bank**: visualization, writing – review and editing. **Belinda Köchle**: data curation, investigation, writing – review and editing. **Bahtiyar Yilmaz**: writing – review and editing. **Jens Becker**: conceptualization, funding acquisition, investigation, methodology, project administration, resources, supervision, writing – original draft, writing – review and editing.

## Funding

This research has been funded by the Novartis Foundation for Medical‐Biological Research (Grant 22A018) and the Swiss National Science Foundation for the Grant 192763 to T.R.J. Open access publishing facilitated by Universitat Bern, as part of the Wiley ‐ Universitat Bern agreement via the Consortium of Swiss Academic Libraries.

## Disclosure

The corresponding preprint to this article is available at https://www.biorxiv.org/content/10.1101/2025.04.08.647752v1.full.pdf.

## Conflicts of Interest

The authors declare no conflicts of interest.

## Supporting Information

Additional supporting information can be found online in the Supporting Information section.

## Supporting information


**Supporting Information** In the Supporting Information, pairwise SNP distances are shown alongside the distribution of SNP counts across both the livestock and wildlife study population. Additionally, sampled wildlife species and corresponding ESBL‐Ec prevalences are shown including 95% confidence intervals. For every ESBL‐Ec isolate of this study, detected genes conferring antimicrobial resistance (grouped by antimicrobial class) and resistance to quaternary ammonium compounds, as well as presence of plasmids are presented. Table S1: Pairwise SNP distances of extended‐spectrum beta‐lactamase‐producing *E. coli* from Swiss livestock (dairy cows) and Swiss free‐ranging wildlife (various species) isolated from 2021‐2023. Table S2: Distribution of SNP counts in extended‐spectrum beta‐lactamase (ESBL)‐producing *Escherichia coli* isolates from Swiss livestock and wildlife, 2021‐2023. Table S3: Observed ESBL‐*E. coli* prevalence in wildlife by species. *n* denotes the number of sampled animals per species, and ESBL‐positive the number of animals from which ESBL‐producing *E. coli* was isolated. Prevalence is the proportion of ESBL‐positive animals. Binomial 95% confidence intervals (CI) were calculated using the Wilson score method without continuity correction. For species with zero positives, the CI reflects the upper prevalence compatible with the observed sample size. Table S4: Detected genes conferring antimicrobial resistance and resistance to quaternary ammonium compounds in extended‐spectrum beta‐lactamase (ESBL)‐producing *Escherichia coli* isolates from Swiss livestock and wildlife alongside results of phenotypic susceptibility testing using selected antimicrobial drugs, 2021–2023. Table S5: Presence of plasmids in extended‐spectrum beta‐lactamase (ESBL)‐producing *Escherichia coli* isolates from Swiss livestock and wildlife, 2021–2023.

## Data Availability

The data have been deposited to the NCBI with the Accession Number PRJNA1238262. The bioinformatic pipeline, implemented using the Snakemake workflow management system, is available at https://github.com/sheenaconforti/ecoli-wgs/.
